# Application of the Nursing Model Based on Acceptance and Commitment Therapy (ACT) in Improving Mental Health and Quality of Life after Colorectal Cancer Drug Chemotherapy

**DOI:** 10.1155/2021/8142155

**Published:** 2021-12-20

**Authors:** Yali Shi, Hongwei Yu, Jiangyong Miao, Lihui Wang

**Affiliations:** ^1^Gastrointestinal Surgery, Hernia and Abdominal Wall Surgery, The Second Hospital of Hebei Medical University, Shijiazhuang 050000, China; ^2^Nursing Department, The Second Hospital of Hebei Medical University, Shijiazhuang 050000, China; ^3^Internal Medicine-Neurology, The Second Hospital of Hebei Medical University, Shijiazhuang 050000, China

## Abstract

According to the most current cancer impact statistics, third most commonly diagnosed cancer worldwide is colorectal cancer. Colon cancer, in addition to its physical symptoms, has been linked to mental health issues in patients, according to the study. Dealing with colorectal cancer drug chemotherapy may lead to depression and anxiety in some people. Others are affected by the physical and mental condition of undergoing many therapies at the same time. Throughout the process of diagnosis, a large number of colorectal cancer patients report clinically relevant degrees as well as a decline in overall mental wellness. In the majority of cases, colon cancer patients are cured following therapy, but those who have survived the disease confront a medical range, physical, and challenges in society, for a variety of mental and physical problems such as anxiety and depression. First, meditation therapy is to urge patients to address their issues and feelings instead of dismissing them, but in the dispassionate and unbiased manner that defines the attentive state. Both the patient and the treating professional may benefit from this treatment method, since it appears to be a very effective therapeutic strategy. After colorectal cancer treatment, in studies, it has been demonstrated that ACT improves mental health, and Internet search engines such as Web of Science and Google Scholar as well as Dialnet were utilized to conduct a systematic literature There were 19 articles that fit the criteria. This includes a discussion of the ACT's philosophical and theoretical basis, as well as the treatment itself. On the other hand, the study on ACT for enhancing mental health and quality of life is examined. Several of the available trials had serious flaws, making it impossible to establish reliable conclusions about the effectiveness of ACT for improving mental health and quality of life. The study determined that there is only a small amount of data supporting the use of ACT for improving mental health. The aim of this study is the application of the nursing model on improving the mental health of the colorectal patients. In addition, the limits of the current empirical state of ACT are acknowledged, and the importance of further research is highlighted.

## 1. Introduction

Cancer is a major cause of death [1]. Cancer was diagnosed in one out of every fourteen persons in 2012. Eight out of ten cancer victims died within 5 years after diagnosis [2, 3]. As a result of cancer diagnosis and treatment, there is an increase in psychotic disorders. 16.3% and 10.3% of cancer patients in oncological and haematological settings matched the criteria for clinical anxiety and depression, correspondingly, according to a new meta-analysis of 70 studies from 14 countries. There are currently many institutions and experts that agree with Bultz and Carlson's suggestion that cancer patients' anguish be identified as the sixth [4]. Salmon Clark McGrath Fisher released the book in 2015 and also found that distress was associated to reduce immune function and higher mortality as well as poorer quality of life [5, 6]. A cancer diagnosis is followed by five years of physical symptoms and psychological suffering, interpersonal strain, and sexual issues for 20–30% of patients [7]. On the whole, cancer patients report feeling pain in the range of 35–96%. So, it is no surprise that it was among the most often reported symptoms of diagnosis and treatment, and it is also a major. Psychiatry can improve the quality of life for cancer patients by decreasing feelings of depression. Treatment efficacy is varied and typically viewed as low [8], which leaves opportunity for therapeutic improvements to improve the effectiveness of psychological oncology therapies. As a relatively recent psychotherapy method in psychosocial oncology, acceptance and commitment therapy (ACT) may be particularly useful in the treatment of cancer-related pain and discomfort. The theoretical foundation for accept theory is examined in this narrative review. This is followed by an evaluation and discussion of the current evidence on ACT in cancer patients, as well as suggestions for further study.

## 2. Colorectal Cancer and Mental Health

Large intestinal cancer called malignancy of the colon starts in the colon (colon). This final section of the digestive system is called the colon. In general, colon cancer affects the elderly most, although it may affect anyone at any age. Typically, colon cancer begins with polyps, which are benign (noncancerous) cell groupings that develop on the colon's inner wall. A small percentage of these polyps may develop into colon cancer in the long run. It is possible to have little polyps that do not create any problems. Identifying and eliminating polyps before they turn malignant helps prevent colon cancer; therefore, physicians prescribe frequent screening tests as a strategy to avoid the disease colon cancer that can be treated with a combination of surgical procedures and pharmacological therapies including chemotherapy and targeted therapy. Colorectal cancer occurs when colon and rectal cancer merge.

Factors that may increase your risk of colon cancer include the following:

- Older age people: despite the fact that colon cancer affects people of all ages, the majority of patients are over 50 years old. Children have a greater risk of colon cancer than adults, but physicians are unsure.
- African American race: in comparison to other races, African Americans have a higher risk of colon cancer
- A personal history of colorectal cancer or polyps: the risk of colon cancer in the future is higher for people who have had colon cancer or noncancerous polyps in the past.
- Inflammatory intestinal conditions: there are a number of illnesses, such as ulcerative colitis and Crohn's disease that may raise the risk.
- Inherited syndromes risk: inheritable mutations in some genes can significantly raise the risk of colon cancer. Occasionally, colon cancer can be traced back to hereditary genes. Family members with familial adenomatous polyposis are more likely to get colorectal cancer due to hereditary nonpolyposis or Lynch syndrome.
- History of colon: having a blood relative with colon cancer doubles your chance of getting it.
- Diet: colon and rectal cancer may be linked to a normal Western diet heavy in fats and calories but poor in fiber. Researchers have come up with a variety of outcomes. According to several studies, those who consume red and processed meat have a higher chance of developing colon cancer.
- A sedentary lifestyle: exercise can reduce the risk of colon cancer in those with sedentary lifestyles.
- Diabetes: there is a higher risk of colon cancer in those who have diabetes.
- Obesity: comparatively, obese persons have a higher chance of developing colon cancer and dying from colon cancer than people who are deemed normal weight
- Smoking: tobacco users may have a higher chance of developing colon cancer
- Alcohol: drinking too much alcohol might raise your risk of developing colon cancer
- Radiation therapy for cancer: radiation therapy given to the abdomen to treat previous cancers increases the chance of colon cancer

### 2.1. Treatment

Patients' preferences and general health are taken into consideration while deciding on treatment choices and suggestions, which are based on a variety of criteria, including the kind keep an open mind and ask questions if anything is not obvious. Have a discussion with the doctor about the purpose of each therapy and what to expect during the course of the “Shared decision-making” refers to these sorts of discussion. Together, the patient and your doctor decide on therapies that will help to achieve their health objectives. There are a variety of therapy options for colorectal cancer, which need better treatment decisions by learning more about the studies.

Studies have demonstrated that no matter how old the patient is, these different therapy techniques give equivalent advantages. Older individuals, on the other hand, may face distinct; see what effects surgery, chemotherapy, and radiation therapy have on older individuals. All treatment decisions should take these characteristics into account in order to customize the treatment for each patient. There are a number of factors to consider.

- The patient's various medical issues
- The patient's general health
- Possible adverse effects of the treatment plan
- Other drugs that the patient already takes

### 2.2. Physical, Emotional, and Social Effects of Cancer

When it comes to cancer treatment, there are not only physical symptoms and side effects to worry about, but also emotional, palliative care, also known as supportive care, is the process of managing all along with therapies to delay, halt, or eradicate cancer; it is an important element of the care plan.

The goal of palliative care is to improve the patient's quality of life throughout treatment by controlling symptoms and providing nonmedical assistance to the patient. Such care is available to anybody, regardless of age or cancer kind and stage. It is most effective when it was started as soon as possible, following a cancer patient who received palliative care in addition to their cancer therapy that report less severe symptoms, a higher quality of life, and a higher level of satisfaction with their treatment. Palliative treatments come in a variety of forms, including medication, dietary modifications, relaxation methods, emotional and spiritual support, and palliative therapies including chemotherapy, surgery, and radiation therapy. After treatment, we may be asked to explain the symptoms and side effects, as well as to answer questions regarding them. Be careful to let their healthcare provider know if you are having any problems with their treatment. Healthcare providers can treat symptoms and side effects more promptly if they know about them in advance to keep their self from becoming more significant (Table 1).

According to several research, the incidence of anxiety and depression symptoms in CRC patients throughout the acute survival phase, which is defined as the first 1-2 years after diagnosis, CRC patients reported moderate to severe depression symptoms on the Center for Epidemiologic Studies Depression Scale-8 (CES-D-8), according to a U.S. research published in the Journal of Clinical Oncology. A study done in Australia six and twelve months following a CRC diagnosis found a low incidence of severe anxiety and depression symptoms on the Brief Symptom Inventory-18 (BSI-18) (range = 7-8%). 19% of Southern England patients diagnosed with CRC in the past year had severe anxiety, and 14% had significant depressive symptoms, according to the Hospital Anxiety and Depression Scale (HADS). There has not been much research done on anxiety and sadness symptoms during the acute survival. A handful of them are discussed on this section. According to a study in Taiwan, anxiety and sadness worsened one month following a CRC diagnosis, before reverting to baseline levels three and six months later. An American and Canadian study found that while depressive symptoms did not change, they did drop before and after CRC surgery. Within 12 weeks of diagnosis, Hong Kong researchers administered HADS to CRC patients along with follow-up visits at 3 and 12 months. Overall, patients demonstrated a robust trajectory (65–67%), with recovery (13–16%) or delayed distress (10–13%) being the most common outcomes. Chronic distress was seen in a small percentage of individuals (7–9%). As a result of these research studies, it appears that the majority of patients having anxiety and depression symptoms that are within the normal range during the acute survival cancer-specific distress (e.g., anxiety and posttraumatic stress symptoms) were not assessed in these trials; however, it has been shown to be higher in certain patients.

## 3. Nursing Models in Practice

Here, nursing models and theories are used to demonstrate how they may be used in nursing practice. As a result, the writers of the applications defined the applications using different terminologies.

1. Based on medical problems
2. Based on nursing based on human development, fields of practice
3. Based on nursing interventions or nursing roles

These models serve as a guide to patient treatment and a broad frame of reference that links the structural environment to patterns of behavior and relationships within an organization. One such approach is the Orem self-care nursing model. According to Seedhouse (2000), self-care is described as the actions that individuals participate in to sustain life, health, and well-being in the Orem self-care model of nursing. Self-care agency is one of the major concepts of Orem's paradigm (usually the individual, but in cases of children, it can be parents or caretakers) [15].

## 4. Implementation of Act Theory Based on the Nursing Model

But despite the terrible impacts of cancer, the number of cancer survivors continues to increase. A number of cancer patients also suffer from posttraumatic stress disorder (PTSD) in addition to anxiety and despair. There are a number of factors that influence the quality of life of cancer survivors, including psychological morbidity, more severe physical symptoms, functional impairments, and treatment nonadherence [16]. More than one behavior can be changed to improve health and decrease healthcare expenses. Several health behavior modification therapies, including those that encourage healthy diet and exercise behaviors, have helped to decrease cancer's damaging effects. More research is needed to reach the huge number of cancer survivors. The use of nonfeasible (mediated) intervention delivery can reach a wide range of individuals. Alternatively, health services and other organizations that regularly offer telephone information and help lines might use phone distribution as one of the most accessible mediated channels. As part of the “Can Change” clinical study, survivors of colorectal cancer were compared to those who had undergone standard treatment. Both physical activity and nutritional habits as well as body mass index have seen significant changes in the past several years, as has overall physical well-being and psychological outcomes (posttraumatic development); the Can Change intervention improved cancer-specific quality of life at 6 and 12 months [17]. Studies have demonstrated that theories-based health behavior modification therapies are more successful than nontheory-based treatments. According to the health behavior theories, behavior is affected by individual, intergroup, and group/organizational/community-level. There are a variety of health behavior theories that have been used: transtheoretical model, social cognitive theory, health belief model, and theory of reasoned action/theory of planned action. By employing a variety of cognitive transformation strategies, these theories aim to alter problematic attitudes and cognitions directly (e.g., goal setting). ACT-based treatments were employed for the first time in the Can Change study to enhance a variety of health behaviors among cancer survivors. “Psychotherapy,” a science-based third generation cognitive behavioral therapy, is defined as “the ability to participate in the present moment more completely as a conscious human being and the ability to modify or persevere in behavior, when doing so, it accomplishes desirable outcomes.” This is because ACT believes that cognition is real. Unlike other theories of behavior modification, it focuses on changing the person's connection with cognitions, whereas other theories of behavior modification attempt to change the person's interaction; in order to modify one's behavior, one must make a conscious decision to act in accordance with personal ideals. Internal impediments to establishing and maintaining positive health behaviors include distress and self-defeating beliefs. Consequently, by encouraging connection and commitment to personal values associated with self-management of positive health behaviors, ACT extends existing intervention techniques. Cancer, chronic pain, diabetes, epilepsy, smoking, and obesity have all been shown to benefit with ACT treatments in several trials. As a result, ACT therapies have been shown to be effective. Our research has to be done on a larger scale. Physical activity and diet modification intervention studies have been conducted during the past decade using telephone delivery of information on both topics. In order to generalize these results to colorectal cancer or other cancer survivor groups, we must take into account that each cancer is distinct in terms of its natural history and pathology, as well as in terms of its treatment procedures and side effects. The focus of the earlier study was on quality of life and health behavior results, as opposed to psychological consequences. While this study is part of a multimodal health behavior modification program called Can Change, it also examines cancer patients' misery and quality of life, along with posttraumatic development as well; for colon cancer survivors, there is a link between posttraumatic development (PTD) and spirituality. Examining the impacts and functions of actions such as accepting oneself and paying attention, the book also examines that a number of prior ACT therapies have demonstrated that these two aspects of the model have a favorable impact on psychological functioning. This multimodal health behavior intervention was designed to enhance psychosocial outcomes (posttraumatic development and spirituality, coupled with distress) and cancer-specific quality of life in addition to greater gains in cancer-specific outcome measures (acceptance and mindfulness). Changes in the ACT process factors should have an impact on psychosocial consequences and quality of life [18].

## 5. Overview of ACT

**Figure 1:**
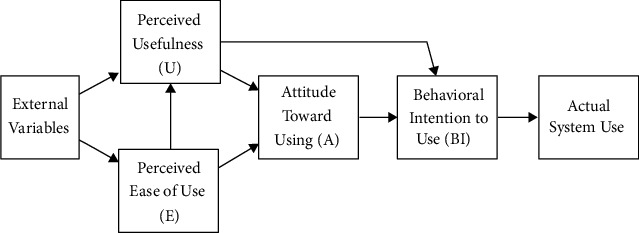
Overview of ACT theory.

Recent years have seen a rise in the popularity of acceptance and commitment therapy (ACT). According to the year 1999, ACT has grown more popular as a mindfulness- and acceptance-based treatment [19], and ACT paradigm has been used in more than 100 randomized controlled trials (RCT) [20]. In these randomized controlled trials, the effectiveness of ACT has been demonstrated for a wide range of mental health issues, including anxiety [21], substance abuse, psychosis [22], aggression, and treatment-resistant psychopathology [23]. Conversely, ACT has become increasingly widely utilized in behavioral medicine, where it is used to treat a variety of illnesses [24]. People with chronic medical conditions can benefit from ACT by reducing their stress and improving [25, 26]. As an example, you might give up smoking. The American Psychological Association's Society of Clinical Psychology has awarded ACT “substantial research money” for its effectiveness (Division 12). Caretakers and support personnel may also benefit from ACT. For cancer caregivers, further research is underway on the effectiveness of ACT [27]. To be sure, despite recent advances in treatment, living with a broad variety cancers comes with some degree of physical and psychological suffering. Due to rising discomfort, the American Cancer Society does not accept the notion that “healthy norms are the norm.” It is one of the ACT's basic principles to “meet the present moment completely and without unnecessary defense—as is and not as it claims to be” [28]. There are three key components of psychological flexibility that contribute to successful adjustment when facing obstacles such as cancer diagnosis or treatment or advanced disease. These are (1) being present, (2) being open, and (3) doing what is important according to the ACT model of psychological flexibility (Figure 1).

It is important to pay attention to current experiences, whether private (thoughts and feelings) or public (events, conversations) or both, in meditation and mindfulness-based interventions (five senses experience such as what can be seen, heard, and touched, such as watching tree branches sway in the wind, hearing the purr of a well-loved cat, or feeling the soothing touch of a partner). No matter how much private and public experiences vary over time, the ACT exercises foster awareness of the “observing self”—the part of oneself that is able to observe what it is seeing. As a result of a catastrophic sickness, a stable and consistent self-perception is a great way to cope with big changes in your profession, relationships, or functioning as emotions develop and fade. ACT fosters psychological engagement. Many people will express their feelings when a medical test finds or removes metastases. It is possible that the pain will fluctuate, but if you are sensitive to emotions, you should have fewer secondary reactions (e.g., distress in response to strong stimuli). As long as they do not seek to change the content or frequency of their presentation in the mind, ideas are categorized. A terrible idea may be more difficult to resist or escape because of the contradictory implications of suppressing thought. On account of the “rebound” effect, someone who tells himself “do not think about cancer coming back” may find himself thinking about it more frequently over time [28]. In line with ACT, individuals are encouraged to explain their personal values and take action based on them. There are many sectors of society where values may be utilized to guide behavior. A person's relationships with others as well as his or her employment are all to understand and clarify the person's views since ACT allows them to withstand difficult situations while making decisions that are consistent with their values [29]. Values-consistent patterns of behavior might include eating healthily and exercising frequently over the course of several weeks or months and then returning to values-based action if a lapse has occurred [30]. After cancer diagnosis and treatment, values-consistent activities and behaviors may alter if symptoms such as pain or tiredness restrict physical mobility or if the disease is in accordance with the patient's energy level and talents at any given time, major or minor acts can be done. However, even in the most difficult and limiting circumstances, it is always possible to take action in the direction of what is meaningful, and that taking action in the direction of what is meaningful improves and meaningfully enrich the lives of all parties involved. Following are the three steps of the ACT approach for coping with life's problems: As a result, you have reached a stage where you are comfortable with your feelings and ideas, while still according to Moran (2013), it can be utilized at any point in the cancer journey.

## 6. Study Conditions

### 6.1. Usual Care

Four educational handouts prepared by the Cancer Council Australia were given to 70 participants who received standard treatment before they began the research. The pamphlets covered topics such as understanding colorectal cancer and decreasing cancer risk. Participants were also provided a quarterly study newsletter, and they were contacted for all follow-up evaluations in order to increase participant retention. The consent was received from the volunteers. During these therapies, the patient can undergo chemotherapy.

### 6.2. Health Coaching

In existing methodologies [29], six months of health counselling over the phone, a member handbook, postcards of encouragement, an activity tracker, and a quarterly study journal were all part of the intervention. ACT processes connected to lifestyle behaviors (values, awareness, systematic desensitization, acceptance, and committed action) are given specific attention [30], health practices in accordance with Australian guidelines [31, 32] as well as personal objectives) throughout the phone sessions. It employed ACT tactics and problem-solving techniques, action planning/goal setting, and an evaluation of health practices as intervention methods. Participants in the study were not charged for the telephone consultations. All licensed coaches with graduate degrees in health promotion, nursing, psychology, or psychology had to complete a six-week study-specific training course on colon cancer incidence, treatment, symptoms, and results, ACT [33]. All phone conversations were also recorded, with the exception of a limited sample that was analyzed to ensure that the intervention procedure was working properly. Research scientists met with Health Coaches on a weekly basis to discuss their findings. In the participant handbook, there was also a table for self-monitoring of health behaviors. Between telephone sessions, participants received encouraging postcard reminders and a Yamax SW700 Multifunction Digi-Walker Pedometer to encourage physical activity [31].

### 6.3. Measurement

Most demographic and comorbidity data (such as age and gender) were collected using computer-assisted telephone interviewing, while the majority of clinical data (such as cancer type, time since diagnosis and treatment, and cancer stage) were collected using records from the Cancer Registry of Canada. In a self-assessment questionnaire at six months, participants assessed their satisfaction with the intervention and the study materials. In two questions, participants were asked to rate the handbook and the level of support they received from the health coach (5 = “excellent” to 1 = “poor”). Two questions were asked if Can Change met their expectations and helped them achieve their goals (4 = “yes, absolutely” to 1 = “no, certainly not”). Participants were asked to assess their overall satisfaction Can Change (4 = “very satisfied” to 1 = “very dissatisfied”) in one question.

## 7. Real-Time Study Characteristics

Among the six investigations, breast cancer [34], ovarian cancer [35], colorectal cancer [36], and mixed cancer populations [21, 37, 38] were all used. As an inclusion criterion in three trials, participants were required to be distressed [38]. As a result, in both Roost et al. (2012) and Feros et al.' (2013) study, study participants were solely the parents of children with cancer or cardiac conditions [39]. The parents of children with cancer or who had significant heart surgery were the only ones who were studied, not the patients themselves on average, between five and seven group sessions lasted 1.5–2 hours [21]. As a result, both Rost et al. (2012) and Feros et al. (2013) looked at parents of cancer patients or those who had significant heart surgery [35, 38]. A lot of the class time was spent on metaphors and experiences. Currently, there are just two randomized controlled that we know of [29, 35], in which individuals were randomly assigned to receive either the intervention or the “treatment as usual” condition. There were no controls in our study; therefore, we only looked at cancer patients' parents or metaphors, and experiences took up a lot of class time. However, as the moment, there are just two random. There were also four instructional pamphlets, as well as quarterly bulletins relating to the study's issue area [29].

### 7.1. Outcome Measures

Outcomes were measured at three months, six months, and twelve months. A long-term follow-up was not possible since many of the patients had died by the time the fourth and eighth sessions came around. As a result of this, a measure of distress and psychological flexibility was added in most research. Only one study used the Medical Outcomes Research Questionnaire Short Form to assess physical pain as a result of the intervention. After therapy, the patient reported that she felt “like her former self” according to Karekla and Constantinou [34]. Because the included studies employed different research techniques, the following are some of the prepost cohort studies and randomized controlled trials that have been analyzed. When evaluating the effects of Feros et al., they found substantial advantages with medium-to-large effect sizes [38]. Reductions in anxiety and mood disturbances resulted in an increase in quality The effectiveness of alternative psychotherapy approaches, such as cognitive behavioral therapy (CBT), has been demonstrated to be no less than that of as a result, there must be a pre-and post-therapy period since there were significant improvements in mood disturbances, quality of life, and psychological well-being. The effect sizes ranged from modest to big (*d* = 0.56–01.38): distress and mood. According to their findings, ACT was equally helpful as other therapeutic approaches, such as CBT. Using randomization as a strategy in individuals who were treated with ACT exhibited improvements in depression severity and acceptance, but those receiving standard treatment (a CBT-style approach) did not. Normal treatment diminished psychological detachment, emotional control, and cognitive suppression, whereas ACT therapy dramatically improved them. However, ACT therapy had a greater impact on anxiety and depression symptoms. At both 6 and 12 months, there were positive effects on posttraumatic development and the cancer-specific quality of life subscale of physical well-being [39]. The benefits of the therapy on spirituality and acceptance were only apparent after six months of treatment. It was shown that both the ACT intervention as well as normal care exhibited significant increases in mindfulness and distress, although they did not vary significantly from their other. A second study (Table 2) found that acceptance and mindfulness assessments were indirectly related to posttraumatic development, spirituality, and quality of life six months after therapy (physical well-being).

**Figure 2:**
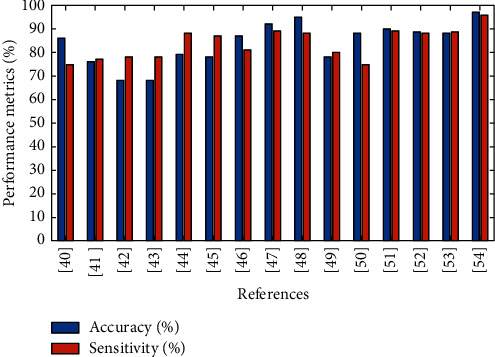
Performance metrics of the existing mechanism over mental health analysis.

From Figure 2, several methodologies have been analyzed, but acceptance and commitment therapy express best performance over mental health analysis and improvement. The suggested model already implemented on different studies will express the high range of accuracy and sensitivity.

## 8. Conclusion

Followed by diagnostics and therapy, a patient's relationship, economic security, and physiological/physical well-being are all affected. As an outcome, cancer patients face a great deal of distress and physical ailments such as pain, and many of them end up suffering from the treatments and diagnosis years thereafter. In cancer settings, psychotherapy has been proven to be useful in addressing depressive symptoms. Mindfulness- and acceptance-based therapies, on the other hand, have attracted increasing attention in recent years. The ACT technique may be especially successful in a medical setting since it emphasizes accepting rather than attempting to minimize symptoms of a basic psychological “disease,” and it has been shown to be helpful in other areas of behavioral healthcare, like chronic back pain. Although ACT has been around more than 30 years, just one study and 5 various projects have looked at its efficacy in people with cancer. Every study looked at a diverse set of patients at different stages of disease progression, many with breast and ovarian cancers, as well as those with colorectal tumors. An improvement in life quality and psychological flexibility, as well as a reduction in disorders like anxiety, mood disturbances, stress, and pain, suggest that ACT could be a valuable psychotherapeutic intervention in cancer settings. In the coming, additional studies in a variety of cancer patients across the illness trajectory should be explored. Considerations for the individual, group, and technology-mediated (for example, phone, web) trials are required. Whenever communicating with parents, significant others, and care professionals, extra-trial considerations must be taken. Clients' and healthcare professionals' perceived barriers, as well as institutional obstructions, should be addressed in the execution of preventive interventions.

## Figures and Tables

**Table 1:** Comparative mental health analysis.

Reference	Sample demographics at baseline	Cancer site and stage	Study design	Theoretical framework	Mental health measures	Results
[9]	N = 542, 57% male, mean age = 71 years, France	63% colon cancer, 37% rectal cancer, 41% stage I, 26% stage II, 19% stage III, 2% stage IV, and 12% unknown	Cross-sectional, population-based, case-controlled (N = 1,181 controls), surveyed at 5-, 10-, and 15-year postdiagnosis	None	SF-36 : MCS, EORTC QLQ-C30: emotional functioning scale, STAI	Mental health and anxiety were not significantly different between cancer survivors and noncancer controls
[10]	*N* = 1,703, 60% male, 71% aged between 60 and 80 years, Australia	Type of CRC not reported, 55% stage 0, I, or II, 35% stage III or IV, and 11% unknown	Longitudinal, surveyed at 5, 12, 24, 36, 48, and 60 months postdiagnosis, population-based	None	BSI	During the 5-year research period, 32–44% of participants reported significant levels of psychological discomfort. According to the study's findings, three distinct distress trajectories were found, including continuous low distress (19%), medium discomfort that varied between time points (30%), medium distress that rose progressively over time (39%), and (13%). Distress was mentioned more frequently by males than when it came to males in distress, they tended to be younger, with less education, a weak social network, and advanced
[11]	*N* = 339, 55% male, mean age = 71 years, Israel	Type of CRC not reported, 18% stage 0 or I, 62% stage II, and 20% stage III	Cross-sectional, surveyed between 2- and 6-year posttreatment	None	BSI, IES, MAC	Survivors who were single and unmarried reported the highest levels of anxiety and help married and unmarried survivors have similar levels of family support, but higher family support was exclusively associated with decreased suffering among married survivors
[12]	*N* = 439, 57% male, mean age = 65 years, Germany	59% colon cancer, 41% rectal cancer, 51% local, 31% regional, 17% distal, and 1% unknown	Longitudinal, surveyed at 1-, 3-, 5-, and 10-year postdiagnosis, population-based, case-controlled (*N* = 2,028 controls)	None	EORTC QLQ-C30: emotional functioning scale	Patients who had been diagnosed with cancer had significantly poorer emotional functioning at 1-, 3-, and 10-year postdiagnosis compared to controls; however, the differences were not clinically significant (>10 points). Comparing younger survivors (age 60) to older survivors (age 70 at diagnosis), younger survivors (age 60) reported substantially poorer emotional functioning 1 and 3 years after diagnosis.
[13]	*N* = 491, 62% male, mean age = 72 years, 76% non-Hispanic White, USA	100% rectal cancer, 53% local, 41% regional, 1% distal, and 5% unknown	Cross-sectional, surveyed at least 5 years postdiagnosis, case-controlled: ostomies (n = 246 cases) vs. anastomoses (n = 245 controls)	None	Modified COH-QOL-ostomy, SF-36 version 2: MCS	As a result of their ostomies, ladies with anastomoses reported a worse psychological well-being; there was also a higher rate of depression among male and female survivors who had ostomies compared to those who did not.
[14]	*N* = 1,419, 53% male, mean age = 70 years, Netherlands	59% colon cancer, 41% rectal, 33% stage I, 38% stage II, 26% stage III, 2% stage IV, and 1% unknown	Cross-sectional, surveyed at an average of 8 years postdiagnosis (minimum of 5 years postdiagnosis), population-based, case-controlled (*N* = 338 normative population controls)	None	HADS	Anxiety symptoms were recorded by 20% of survivors, whereas depression symptoms were reported by 18%. Anxiety levels in survivors were higher than those in the normative group when using a stricter cutoff point of less than 11. Depressive symptoms were higher in survivors than in the normative population when using a stricter cutoff of less than 11

**Table 2:** Comparison over existing methodologies.

Therapies	Accuracy (%)	Sensitivity (%)	Mean square
Compassion-focused therapy [40]	86	75	12.567
Individual supportive therapy [41]	76	77	7.98
Supportive group therapy [42]	68	78	11.542
Cognitive processing therapy [43]	86	79	15.86
Cognitive behavioral therapy [44]	79	88	16.90
Interpersonal therapy [45]	78	87	19.78
Dialectical behavioral therapy [46]	87	81	20.533
Intensive short-term dynamic psychotherapy (ISTDP) [47]	92	89	21.90
Moderated online social therapy (MOST) [48]	95	88	25.97
Music therapy [49]	78	80	15.880
Animal-assisted therapies (AAT) [50]	88	75	26.12
Mindfulness-based cognitive therapy (MBCT) [51]	90	89	17.864
Mindful self-compassion (MSC) [52]	93	90	25.80
Horticulture therapy [53]	88	92	26
Acceptance and commitment therapy [54]	97	96	28.733

## Data Availability

The data used to support the findings of this study are included within the article and are available from the corresponding author upon request.
